# Comparison of Two Versus Three Bilateral Botulinum Toxin Injections Prior to Abdominal Wall Reconstruction

**DOI:** 10.3389/jaws.2023.11382

**Published:** 2023-06-09

**Authors:** Allard S. Timmer, Faduma Ibrahim, Jeroen J. M. Claessen, Carolin J. Aehling, Tom C. P. M. Kemper, Martin V. H. Rutten, Marja A. Boermeester

**Affiliations:** ^1^ Department of Surgery, Amsterdam UMC, University of Amsterdam, Amsterdam, Netherlands; ^2^ Amsterdam Gastroenterology and Metabolism, Amsterdam, Netherlands; ^3^ Department of Anesthesiology, Amsterdam UMC, University of Amsterdam, Amsterdam, Netherlands

**Keywords:** treatment, hernia repair, botulinum toxin, ventral hernia, abdominal hernia

## Abstract

**Background:** Intramuscular injection of botulinum toxin A (BTA) induces a temporary muscle paralysis. In patients with a ventral hernia, preoperative injection of BTA in the muscles of the lateral abdominal wall (LAW) leads to thinning and lengthening of these muscles, making fascial closure more likely. In many hernia centres, treatment with BTA prior to abdominal wall reconstruction has therefore become standard care. However, evidence on the optimal BTA strategy is lacking.

**Methods:** In this single-centre retrospective study, we analysed a consecutive cohort of ventral hernia patients that underwent bilateral BTA injections prior to abdominal wall reconstruction with available CT before and after BTA. We only included patients that were treated with exactly 600 units of Dysport®, diluted into 120 mL of saline, via either two- or three injections on each side into all three LAW muscle layers. The primary outcome was the change in LAW muscle length and thickness, comparing CT measures from before BTA and 4–6 weeks after the injections.

**Results:** We analysed 67 patients; 30 had received two injections bilaterally and 37 had received three injections bilaterally. Baseline data showed no significant differences in LAW muscle thickness or length between groups. In both groups, the median LAW muscle thickness decreased with 0.5 cm (*p* < 0.001). The LAW muscle length increased with 0.9 cm (*p* = 0.001) and 1.2 cm (*p* < 0.001) in the two- and three bilateral injection group, respectively. The BTA-induced changes in LAW thickness and length were not significantly different between both groups (*p* = 0.809 and *p* = 0.654, respectively).

**Discussion:** When using the exact same dosage and distribution volume of BTA in patients with a complex abdominal wall defect, two injections bilaterally in the lateral abdominal wall muscles are as effective as three injections bilaterally.

## Background

The aim of abdominal wall reconstruction for ventral hernia is to close the abdomen by re-approximating the fascial edges. Preferably, the abdominal wall defect is completely closed with native tissue and a mesh is used to reinforce the repair. A bridged repair—in which the defect is not completely closed with native tissue but bridged with mesh - is associated with a high risk of hernia recurrence and should be avoided [1]. In patients with a large hernia defect or with a large amount of peritoneal content residing outside the abdominal cavity (loss of domain), it may be difficult or even impossible to close the abdomen without the use of additional strategies. Component separation techniques (CST) are surgical procedures that involve dissecting one of the lateral abdominal wall (LAW) muscles from the other two by transection of muscle or fascia, thereby reducing the overall contractile force and providing medialization of the fascia. However, because CST pose a serious risk for postoperative wound complications by adding more wound surface, they should only be performed when strictly indicated [2].

An alternative to the invasive surgical CST is the preoperative injection of botulinum toxin A (BTA), also known as chemical CST. When injected into muscle tissue, BTA causes a temporary muscle paralysis. The injections are minimally invasive, preclude the need for surgical disruption of healthy tissue, and have an effect on all injected muscle layers. Its use as preoperative tool for abdominal wall reconstruction was first described in 2009 [3]. Since, several studies have shown that this pretreatment is safe, and provides thinning and elongation of the LAW [4, 5]. Therefore, in many hernia centres, the preoperative injection of BTA has become standard procedure in the prehabilitation process of patients with a large hernia and/or large loss of domain (LOD). Nonetheless, a recent systematic review found that published studies use different types and dosages of BTA, different injection volumes, and a different number of injections [6]. Large heterogeneity in the described treatment regimens and injection techniques makes comparison between studies difficult. In order to achieve some standardization, studies that compare one specific variable are needed. The aim of this study was to investigate if—using the same type and dosage, and dilution volume—two BTA injections bilaterally are as effective as three injections bilaterally, by comparison of its effect on LAW muscle thickness and length on CT imaging.

## Methods

### Study Design

This was a single-center retrospective study. The medical ethics committee of the Amsterdam UMC Location University of Amsterdam, waived the necessity for a formal approval of this study. All patients gave informed consent for use of their data. We report according to the STROBE statement [7]. We previously reported on a subset of patients that are included in this study [8].

### BTA Protocol

In our hospital, we use BTA as a prehabilitation technique for hernia repair. Indications for BTA are a large hernia diameter (>10 cm) and/or large loss of domain (>15%–20%), or substantially retracted LAW muscles (subjective assessment). Our original injection protocol was standardized and consisted of six ultrasound guided BTA injections - three injections on each side—evenly distributed between the lower costal border and the anterior superior iliac spine, on the anterior axillary line. The aim was always to inject at these six locations, and into all three muscle layers (external oblique, internal oblique and transversus abdominis muscles), but deviations occurred due to patient comorbidities, presence of a stomata, fistulas, wounds or distorted anatomy. Per patient we used 600 units of abobotulinumtoxin A (Dysport^®^), diluted in 120 mL of saline 0.9%. All injections were performed by three dedicated anaesthesiologists (MR, CA, TK).

During the years of experience, we noticed that if we performed the most cranial injection first followed by the most caudal injection, the solution had already spread through the muscle, being visible on ultrasound at the location where the middle injection still was to be performed. The increase in muscle thickness, due to the distributed injection fluid of the first two locations, made it more difficult to find an appropriate and effective spot to deposit the third injection. In addition, we observed that the level of discomfort was associated with the number of injections. Therefore, in February 2021 we reduced the number of bilateral injections from three (six in total) to two (four in total). These two injections were also evenly distributed on the anterior axillary line. Of note, all other variables, being BTA type and dosage, volume of the solution, and injection into all three muscle layers, remained the same. We did not use progressive pneumoperitoneum.

### Patient Selection

All patients with a ventral hernia since October 2018 who were [1] treated via either two or three bilateral BTA injections prior to abdominal wall reconstruction and [2] with availability of CT imaging before BTA treatment and 4–6 weeks after BTA were eligible for inclusion. Patients who were treated with another dosage (other than 600 units), volume (other than 120 mL) or selective muscle injections (other than all three muscle layers) were excluded. As this is a retrospective study, the CT imaging protocol was not standardized for the purpose of this study.

### Data Items

Baseline data include age, sex, BMI, hernia location, presence of a- stoma, fistula, infected mesh, and the number of previous hernia repairs. The complexity of the patients/hernias is described using the modified VHWG and HPW classifications [9, 10]. A previous study indicates that the most pronounced effect of BTA pretreatment is a decrease in LAW thickness and an increase in LAW length [8]. Loss of domain larger than 15%–20% is a frequently used cut-off for preoperative BTA, but so far no study has shown that the injections have a significant effect on LOD. The primary outcome of this study was therefore change in LAW thickness and length. Secondary outcomes were changes in hernia width and abdominal width. Because our surgical practice changed considerably within the last few years (for instance type of mesh used and position, type of CST, and use of the Fasciotens^®^ device) we deliberately chose not to compare fascial closure rates and postoperative outcomes between treatment groups.

### CT Measurements

**FIGURE 1:**
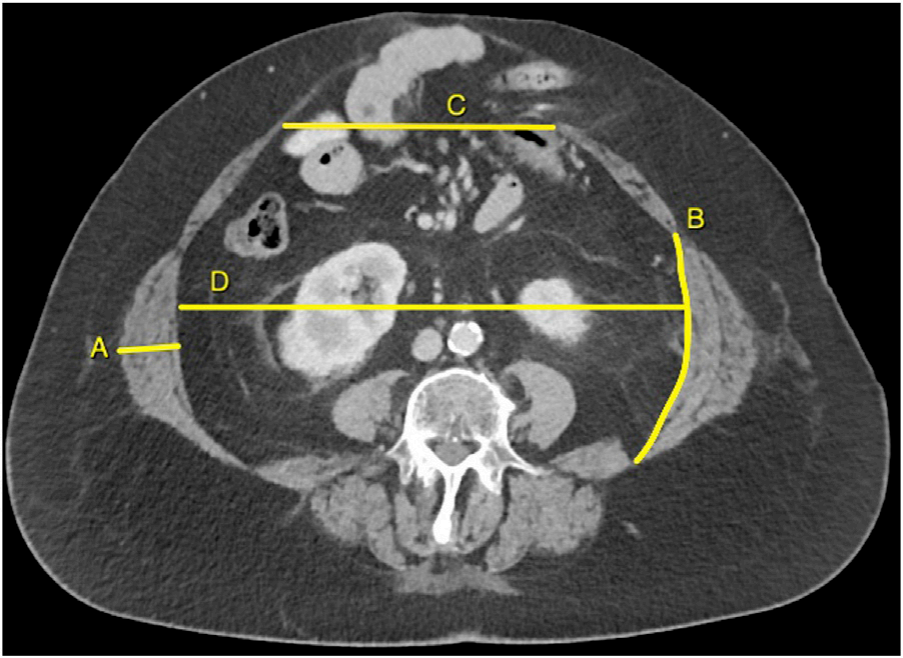
Illustration of how CT measurements are performed. The lateral abdominal wall muscle thickness **(A)** and length **(B)**, and abdominal width **(D)** are always measured on the third vertebral level. The hernia width **(C)** is always measured at the axial level at which the width is maximum.

All CT images were exported as DICOM file, and measurements were performed by one investigator (AT) using an open-source software application (HOROS, www.horosproject.org). Figure 1 illustrates how CT measurements are performed. LAW muscle thickness was measured from the inner edge of the transversus abdominis muscle to the outer edge of the external oblique muscle, halfway the LAW muscle, and perpendicular to the muscle fibers. LAW muscle length was measured along the inner surface of the transversus abdominis muscle, from the lateral edge of the rectus abdominis to the dorsolateral edge of the quadratus lumborum muscle. The LAW muscle thickness- and length were measured individually on each side. Abdominal width was measured as the largest distance inbetween the LAW muscles in a straight line. The LAW thickness, length, and abdominal width were always measured on axial view at the level of the third vertebral level (L3). Hernia width was measured as the largest distance inbetween both rectus muscles, measured at the axial level at which the width was maximum. From patients with a lateral hernia, we only measured the LAW muscle thickness and length from the unaffected side. Hernia width and abdominal width were not measured in these patients. Loss of domain was estimated by using two-dimensional measures of the hernia and abdominal cavity [11].

### Statistical Analysis

Our sample size was limited by the number of eligible patients. Baseline data were summarized and expressed as count with percentage (%) or mean with standard deviation (SD). For every patient we calculated the difference between the pre-BTA and the post-BTA measurements. For LAW muscle thickness- and length we calculated both the overall effect (right and left side combined) and the effect per side. The differences were summarized per treatment group and expressed as median with interquartile range (IQR). Within group differences from before- and after BTA treatment were compared with the related-samples Wilcoxon test. Between groups differences were compared with the Mann-Whitney U test. All tests were two-sided with a *p* < 0.05 considered statistically significant. All analyses were performed with IBM SPSS Statistics for Windows, version 28 (IBM Corp., Armonk, NY, United States).

## Results

### Patient Selection

Between October 2018 and June 2022, we treated 83 patients with BTA injections prior to abdominal wall reconstruction (Supplementary Appendix S1). The effect could not be measured in 10 patients because they had not undergone a CT scan 4–6 weeks after the injections. Another six patients were excluded because they received a different dose, different number of injections, or selective muscle injection.

As such, 67 patients with bilateral BTA in all three muscle layers were analysed; 30 had undergone two bilateral injections, and 37 had undergone three bilateral injections. Baseline data showed no significant difference in sex, age, BMI, presence of- a stoma, fistula, or infected mesh, and the number of previous hernia repairs (Table 1). Relatively, the two injection group contained more patients with a contaminated hernia site (modified VHWG 3): 73.3% versus 43.2% (*p* = 0.021). Consequently, the HPW complexity score was also higher in the two injection group (*p* = 0.016). Before BTA treatment, the LAW muscle thickness and length, hernia width, abdominal width, and loss of domain were not significantly different between groups (Table 1).

**TABLE 1:** Patient data and CT measurements taken before the BTA injections.

	Two bilateral injections (*n* = 30)	Three bilateral injections (*n* = 37)	*p*-value
**Patient characteristics**			
Male sex, no (%)	14 (46.7%)	19 (51.4%)	0.703
Age, mean (SD)	59.6 (12.1)	56.2 (12.1)	0.268
BMI, mean (SD)	29.4 (4.1)	29.2 (4.7)	0.859
Midline hernia, no (%)	25 (83.3%)	32 (86.5%)	0.743
Stoma present, no (%)	10 (33.3%)	8 (20.6%)	0.282
Intestinal fistula present, no (%)	7 (23.3%)	7 (18.9%)	0.659
Infected mesh present, no (%)	7 (23.3%)	3 (8.1%)	0.082
Previous hernia repairs, no (%)			0.751
0	14 (46.7%)	16 (43.2%)	
1	7 (23.3%)	10 (27.0%)	
≥2	9 (30%)	11 (29.7%)	
Modified VHWG, no (%)			**0.021**
1	0	3 (8.1%)	
2	8 (26.7%)	18 (48.6%)	
3	22 (73.3%)	16 (43.2%)	
HPW, no (%)			**0.016**
1	0	1 (2.7%)	
2	5 (16.7%)	18 (48.6%)	
3	20 (66.7%)	13 (35.1%)	
4	5 (16.7%)	5 (13.5%)	
**Baseline CT measurements**			
LAW thickness (cm), median (IQR)			
Right	1.8 (1.7; 2.6)	2.0 (1.7; 2.6)	0.555
Left	1.8 (1.6; 2.3)	2.1 (1.5; 2.4)	0.552
Combined	1.8 (1.6; 2.5)	2.0 (1.6; 2.5)	0.409
LAW length (cm), median (IQR)			
Right	16.7 (13.7; 19.0)	16.8 (14.4; 18.7)	0.618
Left	16.2 (14.6; 19.3)	16.9 (14.0; 18.7)	0.962
Combined	16.4 (14.2; 19.1)	16.8 (14.4; 18.7)	0.793
Hernia width (cm), median (IQR)	10.5 (9.1; 16.3)	13.6 (9.9; 18.3)	0.302
Abdominal width (cm), median (IQR)	26.4 (25.1; 30.0)	28.2 (25.6; 29.9)	0.326
Loss of domain (%), median (IQR)	18.0 (2.9; 33.0)	12.2 (5.8; 18.7)	0.485

Bold is used for outcomes with statistically significant difference.

### Effect of BTA

**FIGURE 2:**
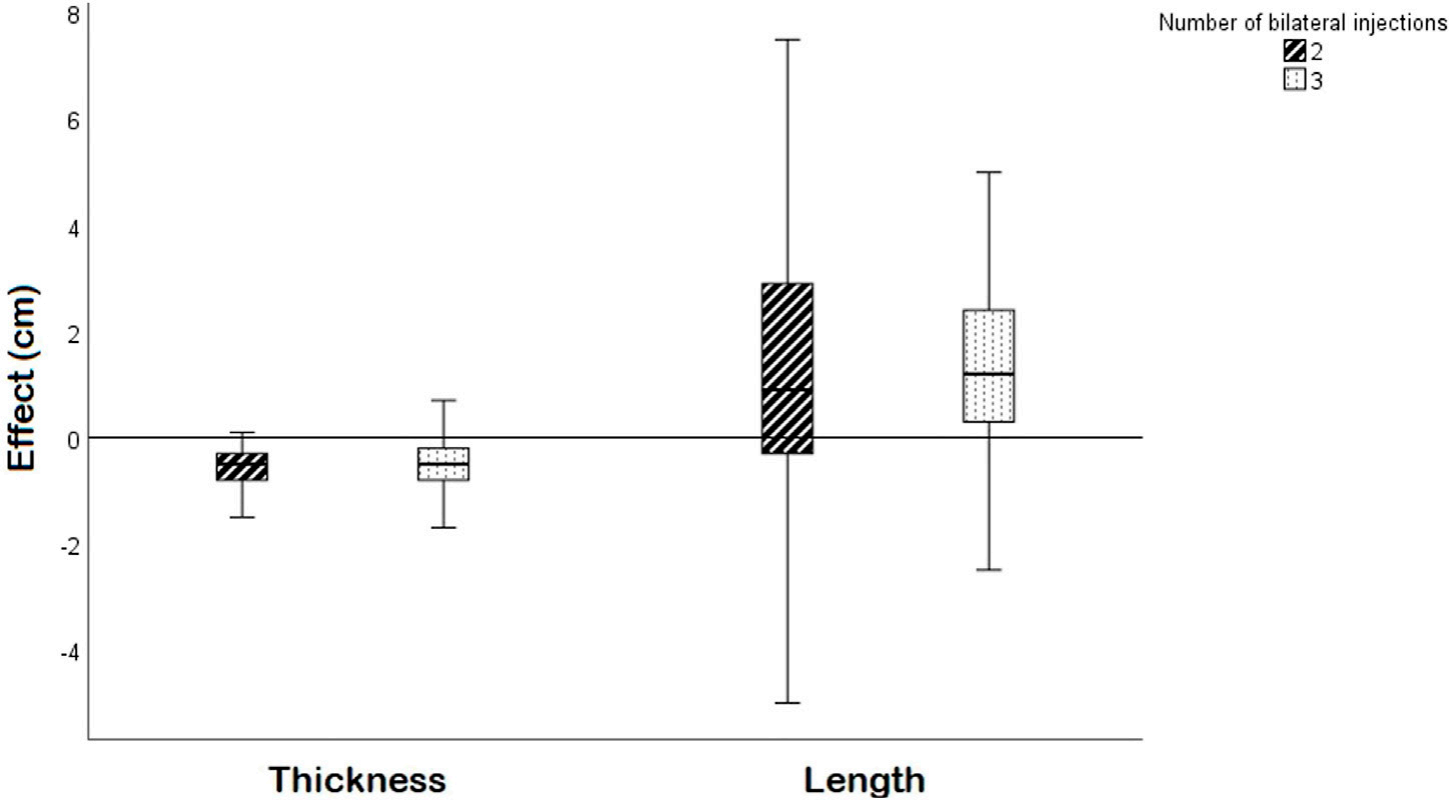
A boxplot showing the effect on LAW muscle thickness and length per side (cm) for the two- and three bilateral injection group. The boxes present the median and interquartile range.

The BTA-induced effects are presented in Table 2. In both groups, the median LAW muscle thickness decreased with 0.5 cm (*p* < 0.001) (Figure 2). The LAW muscle length increased in both BTA groups; with 0.9 cm (*p* = 0.001) and 1.2 cm (*p* < 0.001) in the two- and three injection group, respectively. The BTA-induced changes in muscle thickness and length were not significantly different between the groups (*p* = 0.809 and *p* = 0.654, respectively). Secondly, BTA treatment increased the abdominal width but had no significant effect on the hernia width. These results are comparable for both groups.

**TABLE 2:** The effect of BTA on lateral abdominal wall muscle thickness- and length, hernia width, and abdominal width, compared within groups and between groups.

	Two bilateral injections (*n* = 30)	*p*-value	Three bilateral injections (*n* = 37)	*p*-value	*p*-value for difference between groups
LAW thickness (cm)					
Right	−0.5 (−0.7; −0.3)	<0.001	−0.5 (−0.9; −0.3)	<0.001	0.725
Left	−0.4 (−0.9; −0.3)	<0.001	−0.6 (−0.8; −0.2)	<0.001	0.978
Average	−0.5 (−0.8; −0.3)	<0.001	−0.5 (−0.9; −0.2	<0.001	0.809
LAW length (cm)					
Right	0.4 (−0.8; 3.2)	0.141	1.6 (0.3; 2.9)	<0.001	0.378
Left	1.3 (−0.2; 3.2)	0.002	1.1 (0.0; 2.3)	<0.001	0.613
Average	0.9 (−0.3; 3.1)	0.001	1.2 (0.3; 2.5)	<0.001	0.654
Hernia width (cm)	−0.1 (−1.3; 0.9)	0.291	−0.5 (−2.0; 0.7)	0.088	0.615
Abdominal width (cm)	1.3 (0.2; 3.6)	0.002	1.0 (0.0; 2.1)	0.007	0.322

Differences are presented as median change in cm (with interquartile range). The right column presents the statistical test for differences between both groups.

## Discussion

In this study, we found that preoperative BTA treatment in all three lateral muscle layers using two injections on each side is as effective as three injections on each side in reducing the thickness and increasing the length of the LAW muscles. Furthermore, no differences in effect on hernia width between both treatment strategies was seen.

Use of two injections on each side instead of three has two major advantages. Although the injections are generally well-tolerated, one of the most reported complaints is pain during insertion of the needle. The two-injection technique may reduce patient discomfort. Secondly, it may reduce the procedure time.

We previously investigated how preoperative BTA injections affect the dimensions of the hernia, abdomen, and muscles of the lateral abdominal wall, and found that thinning and lengthening of the LAW muscles are its most pronounced effects [8]. Consequently, we chose the effect on the LAW muscles as primary outcome for the present study. It would be very interesting to investigate differences in clinical outcomes, such as fascial closure rates and hernia recurrence. We specifically chose not to do that in this study because our practice has changed considerably with respect to preoperative optimization and perioperative techniques, and clinical outcomes of both groups are therefore not comparable. Two previous observational studies report higher fascial closure rates after BTA pretreatment, however, differences in baseline characteristics and operative techniques may have biased their outcomes [12, 13].

The observed effect of BTA on muscle thickness and length is smaller than in previous studies. A systematic review of four studies found an increase in LAW length of 3.2 cm on each side [6], where we found an increase of 0.9 and 1.2 cm per side, for the two- and three injections group, respectively. Poor reporting of patient- and hernia characteristics in these studies makes it difficult to explain this difference. However, most studies included patients with a large hernia width, rather than patients with a contaminated hernia site. In our study, 57% of patients had a contaminated/dirty hernia site which likely explains the smaller effect of BTA in present study. Another unexpected finding is the difference in increased muscle length between the right and left side in the 2-injections group. We looked at several baseline variables that may influence a botox effect unilaterally, such as presence (or previous) stomas/fistulas, skin/subcutaneous complications, and previous component separations, but found no results that would explanation a left-right difference. Therefore, it is most likely that the observed difference is a chance finding. Finally, we found a decrease in LAW thickness, and increase in LAW length in the majority of patients. However, in some patients an unexpected opposite effect was observed. We investigated if baseline variables affected the treatment effect but found no association. For now we can only conclude that the effect of BTA on abdominal wall muscle parameters varies in individual patients.

Other studies that directly compare different injection techniques are lacking. In search for the optimal treatment strategy, future studies investigating the ideal dosage, injection volume, and timing prior to surgery are needed. Interesting is the study from Elstner et al. that prospectively enrolled 46 patients to undergo either three injections into all three muscle layers (external oblique, internal oblique and transversus abdominis) or three injections into only two muscle layers (external oblique and internal oblique), sparing the transversus abdominis muscle [14]. Using the same dosage of BTA, they find no significant difference in increase of the LAW length between both groups. One possible explanation is that the transversus abdominis is the thinnest muscle of the three and may have a smaller effect on overall lateral contractile force of the abdominal wall. Nonetheless, transverse abdominis release as a component separation technique, in which the transverse muscle is transected medially, has a clear medializing effect on the lateral abdominal wall.

A limitation of this study is its retrospective nature. The baseline difference among the 2-injection versus 3-injection groups in the percentage of patients with a contaminated hernia site, among other unknown factors, may have biased our results. Despite the unavoidable presence of baseline differences, we exclusively selected patients that received the same type of BTA, dosage, distribution volume, injected in all three lateral muscle layers, with the number of injections being the only injection variable that differed among groups. Another limitation is that we did not perform a sample size calculation, and our results may suffer from type two error.

In conclusion, the two-injection technique may be considered as preferred BTA strategy as it is equally effective. The two-injection technique may reduce the procedure time and may decrease patient discomfort.

## Data Availability

The raw data supporting the conclusion of this article will be made available by the authors, on request to the corresponding author.
