# Supplementary figures and images for: Comparison of Two Versus Three Bilateral Botulinum Toxin Injections Prior to Abdominal Wall Reconstruction
Source: J Abdom Wall Surg. 2023 Jun 9;2:11382. doi: 10.3389/jaws.2023.11382 (PMC10831667; doi:10.3389/jaws.2023.11382)

**Supplementary Appendix**

**Supplement 1.** Flow diagram


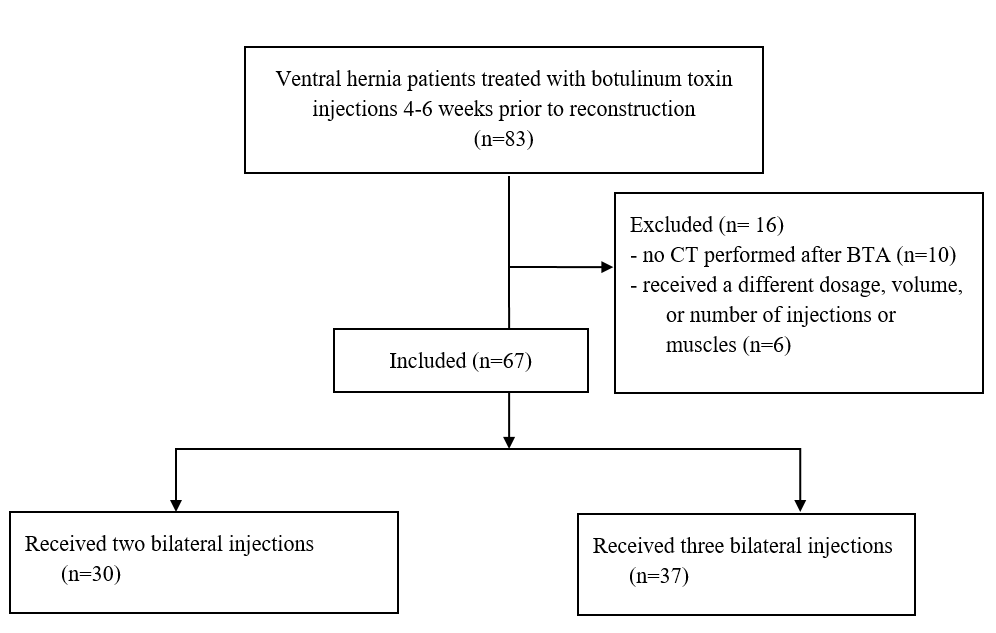

Supplement: Supplementary file 1 [file Table1.docx]
